# Disease burden of herpes zoster in Sweden - predominance in the elderly and in women - a register based study

**DOI:** 10.1186/1471-2334-13-586

**Published:** 2013-12-12

**Authors:** Marie Studahl, Max Petzold, Tobias Cassel

**Affiliations:** 1 Department of Infectious Diseases, The Sahlgrenska Academy at the University of Gothenburg, Diagnosvägen 21, S- 416 85 Gothenburg, Sweden; 2 Department of Medicine, The Sahlgrenska Academy at the University of Gothenburg, Medicinaregatan 19 D, S-413 90 Gothenburg, Sweden; 3 Sanofi Pasteur MSD, Hemvärnsgatan 13, S- 171 54 Solna, Sweden

**Keywords:** Herpes zoster, Hospitalization, Antiviral prescriptions, Mortality, Disease burden

## Abstract

**Background:**

The herpes zoster burden of disease in Sweden is not well investigated. There is no Swedish immunization program to prevent varicella zoster virus infections. A vaccine against herpes zoster and its complications is now available. The aim of this study was to estimate the herpes zoster burden of disease and to establish a pre-vaccination baseline of the minimum incidence of herpes zoster.

**Methods:**

Data were collected from the Swedish National Health Data Registers including the Patient Register, the Pharmacy Register, and the Cause of Death Register. The herpes zoster burden of disease in Sweden was estimated by analyzing the overall, and age and gender differences in the antiviral prescriptions, hospitalizations and complications during 2006-2010 and mortality during 2006-2009.

**Results:**

Annually, 270 per 100,000 persons received antiviral treatment for herpes zoster, and the prescription rate increased with age. It was approximately 50% higher in females than in males in the age 50+ population (rate ratio 1.39; 95% CI, 1.22 to 1.58). The overall hospitalization rate for herpes zoster was 6.9/100,000 with an approximately three-fold increase for patients over 80 years of age compared to the age 70-79 group. A gender difference in hospitalization rates was observed: 8.1/100,000 in females and 5.6/100,000 in males. Herpes zoster, with a registered complication, was found in about one third of the hospitalized patients and the most common complications involved the peripheral and central nervous systems. Death due to herpes zoster was a rare event.

**Conclusions:**

The results of this study demonstrate the significant burden of herpes zoster disease in the pre-zoster vaccination era. A strong correlation with age in the herpes zoster- related incidence, hospitalization, complications, and mortality rates was found. In addition, the study provides further evidence of the female predominance in herpes zoster disease.

## Background

Herpes zoster is a disease caused by reactivation of varicella zoster virus (VZV), which occurs mainly in the elderly population. Studies from Canada and the United Kingdom have demonstrated an incidence rate of herpes zoster over a lifetime of 28- 30% [1]. Complications such as zoster ophtalmicus, bacterial superinfections, and neurological manifestations i.e. meningitis, encephalitis, and particularly post-herpetic neuralgia (PHN) are common [2]. With increasing age, the risk and severity of complications is enhanced, and accompanied by a substantially higher hospitalization rate [3,4]. Several studies have demonstrated a predominance of females contracting herpes zoster [5].

Antiviral therapy has been used for decades to treat herpes zoster. However, although it is effective in decreasing symptoms, studies on such therapy influencing the incidence of the major complication postherpetic neuralgia (PHN), are lacking [6,7]. In 2006, a live attenuated herpes zoster vaccine received marketing authorization in the US and EU after being shown to bring about a reduction in the incidence of herpes zoster and PHN in individuals over 60 years of age in the Shingles Prevention Study (SPS). It was demonstrated that the herpes zoster vaccine reduced the incidence of herpes zoster by 51.3% and of PHN by 66.5% [8]. Recently, a nearly 70% reduction of herpes zoster was found in persons aged 50–59 after immunization with herpes zoster vaccine [9].

The considerable morbidity related to VZV has prompted some countries to implement prophylactic varicella vaccination [10,11]. In Sweden, there is no national pediatric immunization programme against VZV and 98% of Swedish 12-year olds showed serologic evidence of prior VZV exposure according to a study conducted in 1997 [12]. Hence, most adults in Sweden are at potential risk of developing herpes zoster disease, especially late in life as immunosenescence progresses [13] or during immunosuppression.

The herpes zoster burden of disease in Sweden has not been well examined. In order to evaluate the need for a herpes zoster vaccine and to establish a prevaccination baseline of herpes zoster burden in Sweden we conducted a register-based study. The Swedish National Patient Register and the Swedish National Pharmacy Register contain respectively, data on all hospitalizations and all drug prescriptions in Sweden respectively and the Cause of Death Register contains mortality data related to cause.

The present study extracted gender-specific retrospective data from 2006–2010 concerning herpes zoster hospitalizations and drug prescriptions for antiviral medication as well as zoster-related mortality during 2006–2009.

## Methods

### The Swedish National pharmacy register

The Swedish National Pharmacy Register contains information on drugs distributed from all Swedish Pharmacies, the identity of the drug, doses and prices as well as the patient’s gender, age, and place of residence. All prescriptions are linked to the unique Swedish personal identity number. This information makes it possible to calculate both the number of prescriptions and the number of individual patients receiving medication. In addition, the register includes information on the prescribing doctor’s affiliation [14].

### Data extraction from the Swedish National pharmacy register

The following data were collected from the Swedish National Pharmacy Register between January 1, 2006, and December 31, 2010: gender, age, prescriptions of acyclovir 800 mg × 5 or famciclovir 500 mg × 3 or valacyclovir 1 g × 3 in packages for standard herpes zoster treatment during 7 days. These treatment options are in line with national and international recommendations [15,16]. In addition, patient prescription information text was collected whenever available. Since the antiviral drugs mentioned above may be used in the treatment of other diseases than herpes zoster. e.g. primary infections of varicella zoster virus, and in some cases also in herpes simplex infections, prescriptions containing one or more of the following corresponding words in the patient information text were omitted: simplex, genital, mouth, labial, varicella, prophylaxis, and chickenpox. For each specific calender year within the study period, prescriptions were linked to patients and counted only once, although several prescriptions may have been assigned to the same patient.

### The Swedish National patient register

The Swedish National Patient Register covers 99.7% of all public and private inpatient care units dating from 1987. The patient register contains information on gender, age, discharge diagnoses ICD-10 (on the two- and three digit levels), as well as the duration of hospitalization [17]. In Sweden, every citizen has a unique personal identity number [18] and this number is linked to each hospital stay.

In the registers, the number of dropouts and the number of stays with a missing personal identity number are estimated to be less than 1%. The principal diagnosis is missing in 1% of the hospitalizations [19].

### Data extraction from the Swedish National patient register

Hospitalization rates (patients/100,000) and the number of patients hospitalized were collected from the Swedish National Patient Register between January 1, 2006, and December 31, 2010. The ICD-10 codes investigated were: B02.0 herpes zoster with encephalitis, B02.1 herpes zoster meningitis, B02.2 herpes zoster with other complications from the nervous system (ganglionitis, postherpetic polyneuropathy, postherpetic myelitis, trigeminal neuralgia, other postherpetic nervous system involvement), B02.3 herpes zoster ocular disease, B02.7 disseminated herpes zoster, B02.8 herpes zoster with other complications including bacterial superinfection and B02.9 herpes zoster without complication.

In the Swedish National Patient Register, both primary and non-primary causes of all hospitalizations are recorded. In this study, data on herpes zoster as the primary and non-primary cause in hospitalization were collected. However, for herpes zoster with complications and age-stratified hospitalizations, only primary diagnosis cases are presented.

### The Swedish National cause of death register and data extraction

The Cause of Death Register covers approximately 98% of Swedish deaths. The predecessor to the Register was started as early as 1749 and since 1951, it follows the international standard. Since 1997, ICD-10 codes have been utilized for input data. Death cause data related to herpes zoster (ICD-10; B 02) and gender and age of diseased persons have been collected [20] from January 1, 2006, to December 31, 2009.

### Statistics

Descriptive statistics such as annual numbers and rates per 100,000 population are presented. Trends in annual numbers are assessed using linear regression, and rate ratios are estimated and tested for differences between men and women in different age groups. For each specific calender year within the study periods, prescriptions and hospitalizations were linked to patients and counted only once, although several events may have been assigned to the same patient.

### Ethical approval

The study was approved by the Regional Ethical Review Board in Gothenburg (number 206–10).

## Results

### Prescriptions for antiviral medication related to herpes zoster

**Figure 1:**
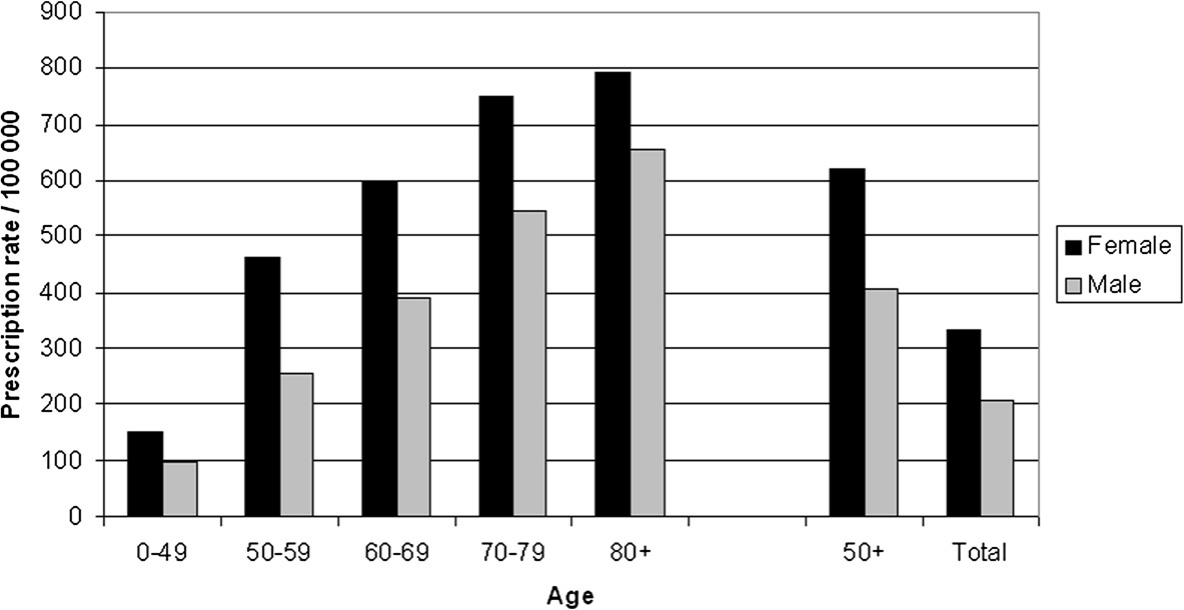
Age-stratified number of male and female patients in Sweden receiving antiviral prescriptions in the dosage used in herpes zoster treatment during 2006-2010.

Annually, approximately 25,500 patients received prescriptions for antiviral medication in packages for standard herpes zoster treatment during 7 days (Table 1). During the study period there was a trend towards an increasing number of annual prescriptions. On average, there was an annual increase of 655 prescriptions for female patients (p = 0.035) and 403 for male patients (p = 0.031) during the study period. Although a number of prescriptions were given to patients between 30 and 49 years of age, there was a progressive increase in prescriptions for patients over 50 years of age (Figure 1). About half of the patients were prescribed acyclovir, while the other half received valacyclovir (data not shown), and a smaller number were prescribed famciclovir.

**Table 1:** Annual number of female and male patients in Sweden receiving antiviral prescriptions in the dosage used in herpes zoster treatment during 2006-2010

**Year**	**Total**	**Females**	**Males**
2006	24 405	15 107	9 298
2007	23 503	14 504	8 999
2008	25 291	15 668	9 623
2009	26 364	16 313	10 051
2010	28269	17480	10789
Reg coeff (p-value)	1058.9 (0.033)	655.5 (0.035)	403.4 (0.031)

For prescription rates, a gender associated pattern with a difference between males and females was discerned in all age groups. The rate was approximately 50% higher for females than in males in the age 50+ population (rate ratio, 1.39; 95% CI, 1.22 to 1.58). The difference was most pronounced in the 50–59 age group, where the prescriptions rates were almost two-fold higher (rate ratio, 1.81: 95% CI, 1.67 to 1.93) for females than for males (Figure 1).

### Hospitalizations related to herpes zoster

The average annual number of hospitalized patients diagnosed with herpes zoster as the primary cause was 636 over the study period. On including patients hospitalized with herpes zoster as both the primary or non-primary cause, the average annual number of patients was 1179 during the same period. The number of patients, the hospitalizations, and the average time of hospitalization showed little or very low variability from year to year during the study period. The average period of hospitalization was approximately 7.5 days for cases with a primary diagnosis and 10 days for cases with a non-primary diagnosis (data not shown).

In the total population, the hospitalization rate for herpes zoster (primary cause) was 6.9/100,000. The corresponding data (primary cause) were 8.1/100,000 and 5.6/100,000 in females and males, respectively. For cases with herpes zoster disease as the primary or non-primary diagnosis, the rate was 13.2/100,000 in the total population.

**Figure 2:**
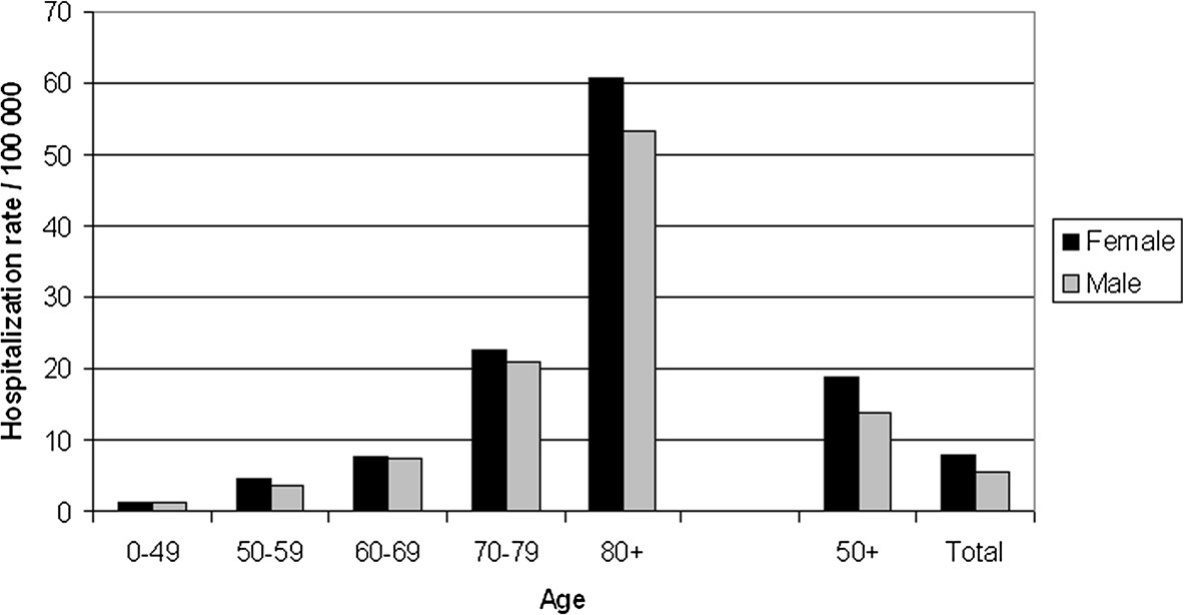
Age-stratified annual hospitalization rates for herpes zoster disease as the primary diagnosis in males and females in Sweden during 2006–2010.

Hospitalizations for herpes zoster disease were rare in individuals under 50 years of age. In the 50-year-old, and older population, the mean annual hospitalization rate (primary cause) for cases with herpes zoster disease was significantly higher for females with 18.8 hospitalizations/100,000 compared to 13.7 hospitalizations/100,000 for males (Figure 2). In persons over 70 years of age the hospitalizations rates were seen to accelerate with a subsequent rapid increase for older age groups. The difference in hospitalization rates between males and females was most pronounced in persons aged 80 years and older (Figure 2).

### Complications of herpes zoster

Herpes zoster with a registered complication was found in 33% of hospitalized females and 24% of hospitalized males, (p < 0.001). For hospitalizations, herpes zoster without complications (B02.9) was the most common diagnosis recorded. The most common complications were from the peripheral and central nervous systems (B02.0, B02.1, and B02.2). Other common complications were herpes zoster opthalmicus (B02.3) and miscellanous complications, including bacterial superinfection (B02.8). Disseminated herpes zoster (B02.7) was rarely recorded (Table 2).

**Table 2:** Average annual percentage and annual number of patients hospitalized (primary diagnosis) in Sweden for herpes zoster disease with or without complications during the study period 2006 – 2010

**Diagnosis**	**Females % (n)**	**Males % (n)**
Zoster encephalitis (B02.0)	2.6 (10)	2.1 (5)
Zoster meningitis (B02.1)	1.8 (7)	2.1 (5)
Zoster with other nervous system involvement (B02.2)	7.8 (29)	5.0 (13)
Zoster ocular disease (B02.3)	8.6 (33)	6.1 (16)
Disseminated zoster (B02.7)	2.4 (9)	2.9 (7)
Zoster with other complications (B02.8)	9.7 (37)	6.1 (16)
Zoster without complications (B02.9)	67.1 (254)	75.7 (195)

### Herpes zoster mortality

**Figure 3:**
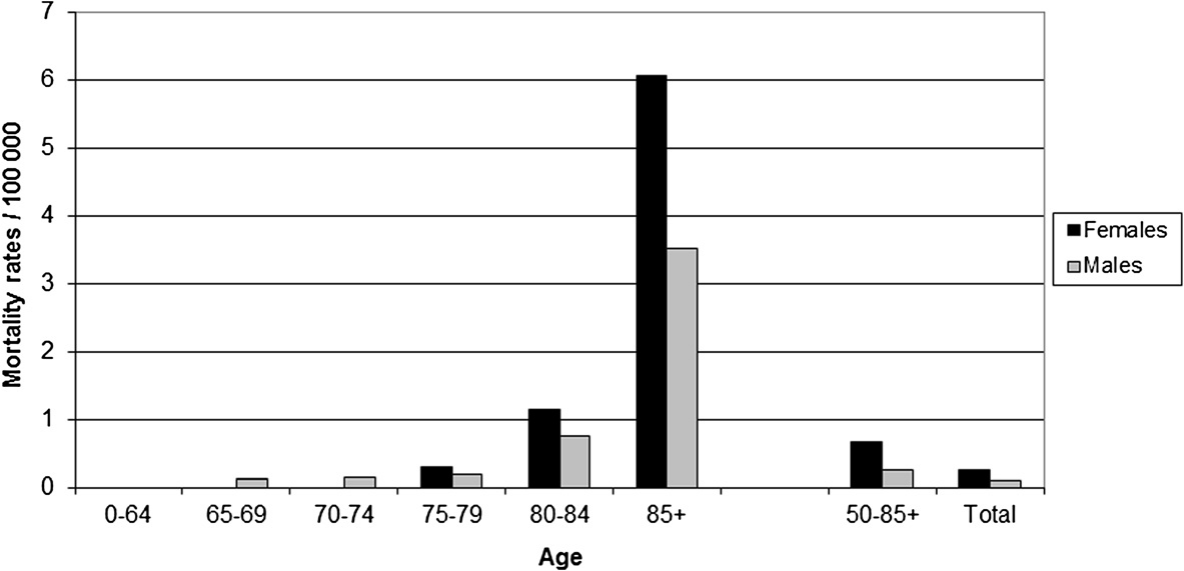
Annual age-specific mortality rate in patients with herpes zoster as the primary cause in Sweden during 2006–2009.

The average annual mortality with herpes zoster as the primary cause during the study period was low and the first cases of death occurred in the age groups 65 years and older. In general, the mortality rates were higher in women than in men. Mortality rates increased with age and the oldest segment of the population had the highest mortality rate. The average annual mortality rate in the age group 85 or older was five-fold higher compared to that in the 80–84 age group (Figure 3). The average annual mortality rate in the age group 50 and older age group was 0.67/100,000 in females and 0.26/100,000 in males. It should be noted that the cause of death is directly related to the herpes zoster disease in the judgment of the treating doctor.

## Discussion

In this study we have estimated the burden of acute herpes zoster by utilizing National Swedish Health Care Registers. The disease burden has been studied using three separate approaches: analyzing antiviral medication prescription data, demonstrating zoster-related hospitalizations, including complications, and herpes zoster-related mortality. However, the study lacks an estimation of the total burden of disease related to VZV reactivation since analyses of prescriptions, hospitalizations, and mortality related to PHN are not included in the analysis. A strength of this study is the solid national data from the Swedish National Patient Register with a very low number of dropouts or lack of principal diagnoses, which are estimated to occur in only one percent of cases, respectively [19]. Primary care visits are not analyzed in this study because no national health care register has been established for such visits in Sweden.

The Swedish National Pharmacy Register provides an exclusive opportunity to analyze prescription patterns since each prescription is linked to the unique personal identity number of the patient. The prescription data may be regarded as an indirect measurement of the herpes zoster incidence and primary care visits in Sweden. This assumption is plausible since, in this study, most antiviral medication prescriptions for herpes zoster were found to be written by doctors in primary care (data not shown). Similar findings were reported in an Australian study [21]. In Sweden, patients under 50 years of age are not recommended antiviral treatment [15] and it was found that approximately 70% of the prescriptions were distributed to patients over 50 years of age. Thus, a theoretical association of the antiviral prescription data with the incidence of herpes zoster is only feasible for the population over 50-year-old population, and older. This results in a national minimum incidence of herpes zoster in Sweden of 621/100,000 for females and 404/100,000 for males in the population age 50 and older, based on antiviral prescriptions. Recently, a review of European studies, focusing on total population incidence of herpes zoster, stated that in the 21 included studies the incidence in all ages was 2–4.6/1000 inhabitants/year which is in line with our results [22]. In the review by Thomas et al., where studies from both Europe and USA were included, the incidence in all ages varied from 3.6 to 14.2/1000 inhabitants/year [5]. However, methodological differences are frequent and direct comparisons of studies must address this circumstance.

The analysis of the prescription data has some inherent uncertainties. Even if precautions have been taken to exclude prescriptions not related to herpes zoster, the data may contain prescriptions for patients who received antiviral treatment in the dosage for herpes zoster for other indications than herpes zoster. However, the Swedish Medical Product Agency does not recommend treatment with dosages compatible with that used in herpes zoster disease except for herpes simplex meningitis [16], a relatively rare disease compared to herpes zoster disease. The possible inclusion of non-herpes-zoster-related patients is a limitation of the study and may result in an overestimation of the prescription rates. On the other hand, the number of patients with herpes zoster most likely exceeds the number of prescriptions, since immunocompetent patients under 50 years of age with uncomplicated herpes zoster are not recommended antiviral treatment in Sweden [15]. In the review by Pinchinat et al. [22], prescription studies were not considered as reliable as prospective studies in health care facilities or retrospective studies of herpes zoster cases identified through the review of medical files when calculation the incidence of herpes zoster. However, an overestimation of cases in our study is less likely since only approximately 72% of the patients diagnosed (ICD -10) with herpes zoster in primary care receive a prescription of antivirals (Dr. Lars Rombo, personal communication).

In the present study, hospital cases with non-primary and primary diagnoses of herpes zoster disease were analyzed. Herpes zoster occurs more often in elderly people, but it is not unusual that hospitalization is the result of other diseases. Thus, herpes zoster may be recorded as a non-primary cause in these cases. At the same time, it cannot be ruled out that patients with PHN might be misdiagnosed as a herpes zoster case in the group of non-primary causes. On adding herpes zoster as a non-primary diagnosis to the analysis, it was estimated that the annual average number of hospitalized patients was 1,179, while the annual average number of patients with a primary diagnosis was 636. However, for the herpes zoster complications and age-stratified hospitalizations, only primary diagnoses were examined to ensure a causal link to herpes zoster.

The disease burden in hospitals is significant, expressed in the rates of hospitalization, and is similar to that in a recent report from France [4]. In the present study, the majority of the hospitalizations occurred in patients over 50 years of age. The increase in hospitalization rates followed a particular pattern and there was a stepwise increase between the age groups (Figure 2). In the group 80 years or older there was nearly a three-fold increase in the hospitalization rates compared to the previous age-group, reaching about 61/100,000 and 53/100,000 in females and males, respectively.

In a recent study from South Korea, it was demonstrated that the hospitalization rates were increasing from 2003 to 2007 [23]. It was speculated that this finding was related to a relative increase in the size of the elderly population along with an increasing number of patients with immune deficiencies. We did not note such an increase in hospitalization rates in our study. However, the annual number of antiviral prescriptions for herpes zoster increased by an average of 1,059 (p = 0.033) during the study period. It is likely that cases of herpes zoster will increase in the coming decades due to a growing elderly population. According to the National Population Statistics in Sweden, the life expectancy in this population is projected to increase from approximately 83.1 to 86.3 years in females and from 79.1 to 83.6 years in males between the years 2009 and 2050.

Herpes zoster complications were recorded in approximately a third of hospitalized female cases while about a fourth of male cases developed complications. Complications stemming from the peripheral and central nervous systems dominated, quite similar to the findings in the recent study in France [4]. There are reports of increased numbers of cases detected after the introduction of VZV quantitative PCR on cerebrospinal fluid samples [24] and this method was implemented during the study period in Sweden. Thus, nervous system involvement may have been underestimated or underdiagnosed in the study. Zoster with other complications, disseminated herpes zoster, and zoster opthalmicus were also quite common, demonstrating the broad spectrum of complications during herpes zoster disease. Bacterial superinfections (which are included in zoster with other complications) with septicemia and phlegmone are serious complications, nearly as common as the nervous system complications.

The predominance of females was one of the major findings in this study and was demonstrated in all the analyses, i.e. the antiviral prescription, hospitalization and mortality rates. The pattern of female predominance was most pronounced for prescription rates where female patients had generally higher rates of prescription of antivirals than males (rate ratio 1.61; 95% CI, 1.57 to 1.65). The female predominance was particularly evident in the 50–59 age group where female patients had generally higher rates of prescription of antivirals compared to males (rate ratio 1.83, 95% CI, 1.68 to 1.99). The pattern of female predominance was also seen for hospitalization rates, where females in all age-groups were significantly more often hospitalized compared to males (rate ratio 1.40; 95% CI, 1.20 to 1.64). In the European review by Pinchinat et al., the incidence rates were systematically higher in females than in men in the included studies confirming the results of this study [22,25-28]. Even though a predominance in females of antiviral prescriptions was demonstrated in several age groups in the present study, it cannot be excluded that this finding is related to a different health care seeking pattern for herpes zoster between the sexes in Sweden [29]. In addition, gender difference in the incidence of herpes zoster might be due to immunological or hormonal differences between men and women. However, this issue is out of the scope of the present study and should be further explored.

## Conclusions

The results of the present analysis demonstrate the strong correlation with age in the herpes zoster- related incidence, hospitalization, complications, and mortality rates in Sweden. In addition, the study provides further evidence of the female predominance in herpes zoster disease. The results of this study also provide a prevaccination baseline for the herpes zoster burden in Sweden. Many countries project a growing elderly population and an increasing number of patients with treatments suppressing the immune system. Hence, adequate strategies need to be established to reduce the burden of herpes zoster today and in the future.

## Competing interests

T.C is an employee of Sanofi Pasteur MSD which is the marketing authorization holder of the herpes zoster vaccine (Zostavax) in Europe. M.S has received an unrestricted research grant from Sanofi Pasteur MSD to fund the present study in part.

## Authors’ contributions

TC and MS designed the study.TC, MS and MP analyzed and interpreted the data and compiled the manuscript. MP was responsible for the statistical analysis. All authors approved to the final version of the manuscript.

## Pre-publication history

The pre-publication history for this paper can be accessed here:

http://www.biomedcentral.com/1471-2334/13/586/prepub
